# Surface Characterization of Bone-Level and Tissue-Level PEEK and Titanium Dental Implant Scan Bodies After Repeated Autoclave Sterilization Cycles

**DOI:** 10.3390/dj12120392

**Published:** 2024-12-03

**Authors:** Syed Saad Bin Qasim, Aqdar A. Akbar, Haneen A. Sadeqi, Mirza Rustum Baig

**Affiliations:** 1 Department of Bioclinical Sciences, College of Dentistry, Kuwait University, Safat 13110, Kuwait; 2 Department of General Dental Practice, College of Dentistry, Kuwait University, Safat 13110, Kuwait; aqdar.akbar@ku.edu.kw; 3 Department of Restorative Sciences, College of Dentistry, Kuwait University, Safat 13110, Kuwait; haneen.sadeqi@ku.edu.kw (H.A.S.); mirza.baig@ku.edu.kw (M.R.B.)

**Keywords:** surface characterization, autoclave sterilization, dental implant, scan bodies

## Abstract

**Background:** Sterilization is required for any biomedical device intended to be used in contact with the human body. Several studies have reported alterations in the bulk and surface properties of such devices after repeated sterilization cycles. These surface modifications may influence other clinical parameters. Therefore, the aim of this study was to investigate the surface and chemical properties of implant scan bodies (SBs) after consecutive autoclave sterilization procedures. **Methods**: The objective was to analyze the scan bodies using Fourier transform infrared spectroscopy (FTIR) and X-ray photoelectron spectroscopy (XPS) for chemical analysis and an optical profilometer to analyze the surface roughness. **Results**: FTIR spectra depicted the appearance of peak at 1741 and 1100 cm^−1^ due to the diphenyl ether band disappearance. The XPS spectra showed alterations in the elemental composition after autoclaving and roughness were significantly reduced in PEEK BL and TL SBs. **Conclusions:** These results indicated that some surface modifications were induced by repeated sterilization cycles.

## 1. Introduction

Scan bodies (SBs) are essential elements of implant-related digital workflow that are attached to implants or laboratory replicas and scanned using intraoral scanner or laboratory scanners. The design software program matches the ultrastructural features of the scan body to an existing library, and by surface matching it positions the digital scan body and hence the digital implant three-dimensionally in the virtual dental arch [1]. Generally, scan bodies have a common ultrastructure that consists of a scan region, body, and base. The scan region and body are typically composed of the same material. However, the base may or may not be manufactured from the same material as the body [1].

Various types of biomaterials have been used to fabricate these scan bodies. Most commercially available SB systems are made up of synthetic polymers, metals, or even composite materials of metals and polymers. Polyether ether ketone (PEEK), titanium (Ti) alloys, and aluminum alloys are commonly used biomaterials [2]. The drawback of using titanium SBs is their light-reflective surface, which is a hurdle in acquiring a proper scan [3]. PEEK is a polyaromatic semi-crystalline thermoplastic polymer [4,5]. It is well known for its use in high-performance engineering applications. It has excellent resistance to chemicals, solvents, and hot water, and it can be continuously used up to 250 °C for a very long duration in air, in comparison to other thermos plastics [6].

Past studies have assessed the effect of reuse of implant impression copings and scan bodies on the accuracy of dental implant impressions; however, research analyzing the surface degradation and chemical compositional changes of implant scan bodies after repeated use and sterilization is rare.

An interesting aspect of this polymer is its ability to be tuned with respect to its mechanical properties depending on the application. This can be achieved by varying the manufacturing temperature [5]. The thermal processing or annealing of the polymer affects the crystallinity of the structure and hence has a direct impact on the mechanical properties. Generally, the yield strength of PEEK increases with an increase in crystallinity. The presence of a diphenyl ketone group is the reason for its high thermal stability as it imparts high oxidation resistance and strength [6]. Some PEEK materials are reinforced with hydroxyapatite or carbon fibers. The fiber content and orientation affected the elastic modulus. However, details of the specific PEEK variants used in the fabrication of SB systems are unavailable [5]. The Young’s modulus and tensile properties of PEEK are comparable to those of human bone and teeth [4]. PEEK has an advantage in terms of light reflection, which is a shortcoming of Ti; nevertheless, it may undergo changes when exposed to thermal processes. In addition, possible alterations of the base due to repeated use or sterilization might affect a scan’s accuracy due to the displacement of the SBs [1]. To the authors’ best knowledge, no available studies in the literature have investigated the surface roughness of scan bodies after repeated sterilization, using optical profilometry.

To avoid deformation, SBs fabricated using PEEK are advised to be used one time only. The cost associated with SBs is considerable, and it may be viable to reuse these components. However, the accuracy of the scans acquired with PEEK SBs after being hand-tightened up to 10 times, without sterilization, has been reported to show insignificant differences. Reports on the effects of repeated sterilization procedures, such as autoclaving, on the physical and chemical properties of commercially available PEEK and Ti SBs are limited [7].

The study objective was to chemically characterize the scan bodies using Fourier transform infrared spectroscopy and X-ray photoelectron spectroscopy, and to perform the surface roughness analysis of the scan body on 16 standardized locations, using an optical profilometer, before and after three consecutive autoclave sterilization cycles. The null hypothesis was there would be no differences in the surface roughness values of the four different scan bodies after repeated sterilizations.

## 2. Materials and Methods

### 2.1. Autoclave Sterilizaiton

**Figure 1:**
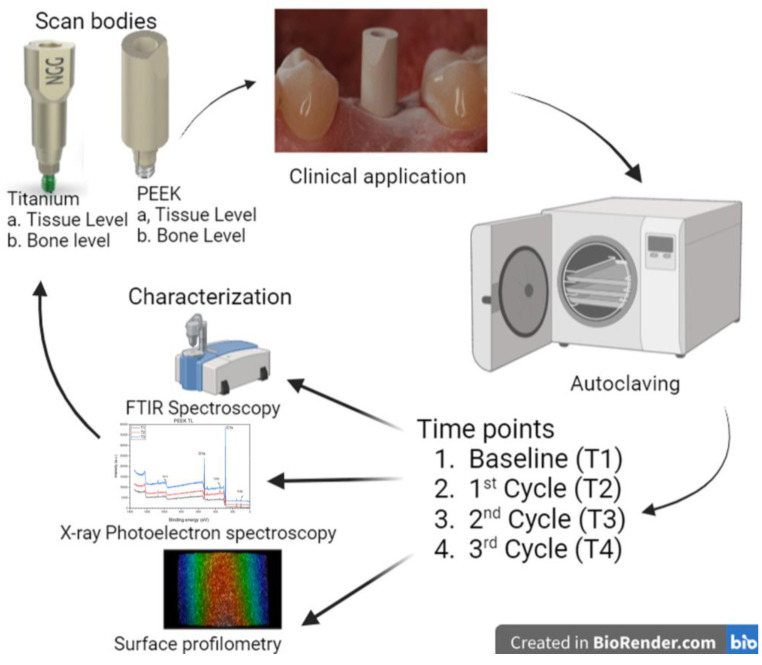
Diagrammatic illustration of the study design showing scan bodies, their clinical application, and the characterization done on them. Image made by using https://www.biorender.com/ (accessed on 10 June 2024).

To perform the steam sterilization cycles, a class B autoclave (Sterlizer LISA W&H Sterilization, Burmoos, Austria) was used. Samples (Table 1) were heated up to 134° Celsius for a 20 min rapid cycle at a pressure level of 210–230 kPa and close to 100% humidity. After each cycle, SBs were characterized and re-sterilized using the same protocol. Figure 1, shows the diagrammatic illustration of the study design.

### 2.2. Fourier Transform Infrared Spectroscopy

Fourier transform infrared (FTIR) spectroscopy was performed using the attenuated total reflectance mode (ATR) (Bruker, Tensor 27 system, Bruker Optics, Ettlingen, Germany, GmbH) equipped with a diamond ATR crystal. Spectra were collected from neat (pristine) Ti BL, Ti TL, PK BL, and PK TL in the mid-infrared region of 400–4000 cm^−1^ at a spectral resolution of 4 cm^−1^. A total of 32 scans were acquired per pixel. A background spectrum was collected prior to analyzing the specimens in order to eliminate any existing peaks of water vapor and carbon dioxide. This background spectrum was taken every 30 min to ensure precise background subtraction. The obtained spectra were processed using OMNIC™ Software, version 8.2.0.387.

### 2.3. X-Ray Photo Electron Spectroscopy

An ESCA lab250xi instrument (Thermo Scientific, Waltham, MA, USA) equipped with a monochromator Al Kα X-ray irradiation source of power 1486.5 eV and charge compensation flood gun had been used in the surface investigation. All spectral lines were corrected with regard to C1s at 284.6 eV. After the vacuum reached ×10^−7^, the specimen was transferred to the analysis chamber for scanning. The scanning and peak fit process was carried out with Thermo Advantage software (v5.956). The full survey spectra were collected on a range of 0–1300 eV at a pass energy, dwell time, and step size of 150 eV, 50 ms, and 1 eV, respectively.

### 2.4. Optical Profilometery

The surface roughness of the specimens was measured using an optical profilometer (Leica Microsystems DCM8, GmbH, Wetzlar, Germany). The surface roughness values were measured before and after each sterilization cycle. The measurement points for surface roughness evaluation were standardized for each scan body. The two bucco-occlusal sharp corners on each scan body in relation to the flat area were used as reference points. Two other reference points were used equidistant from these initially determined locations. Along the entire scan region of the scanbody, at each of the 4 pre-determined points, 4 locations were identified at 2 mm distances occluso-cervically and surface-roughness evaluated, resulting in a total of 16 measurement points for each scan body. The surface roughness (Ra) values of 16 selected points were averaged to provide the mean Ra value for each scan body. The images were acquired using a 10X objective lens. Additionally, the total volume loss (%) was also calculated using the data points. The formula used to calculate volume loss is as follows:

Volume loss (%) = (V_initial_ − V_final_)/V_initial_ × 100

whereby V_inital_ is the volume before sterilization and V_final_ is the initial volume after sterilization.

## 3. Results

### 3.1. FTIR

**Figure 2:**
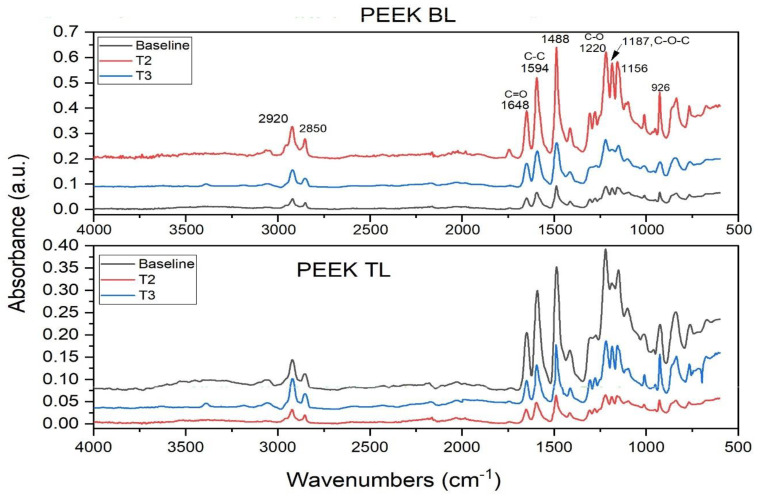
FTIR spectral profile of PEEK bone-level and tissue-level scan bodies. Spectral data shown were collected at baseline (before sterilization) and after each sterilization cycle. The peaks have been identified within the fingerprint region.

**Figure 3:**
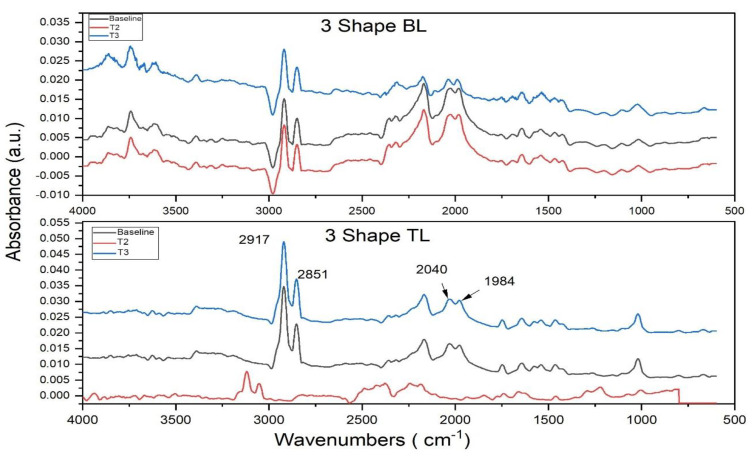
FTIR spectral profile of 3 Shape bone-level and tissue-level scan bodies. Spectral data shown were collected before sterilization as blank and after sterilization cycles. The peaks have been identified within the fingerprint region.

FTIR spectral data of PEEK and Ti scan bodies are shown in Figure 2 and Figure 3. Table 2 describes the spectral changes at specific FTIR peak values. The spectrum of PEEK BL and TL contains a series of absorption bands shown from 500 to 4000 cm^−1^. The absorption band at 3051 cm^−1^ corresponds to C-H stretching vibration for hydrogen attached to aromatic rings. The bands at 2920 and 2850 cm^−1^ are attributed to the alkane -CH_2_ asymmetric and symmetric stretching modes. An increment in the intensity of the bands can be seen after sterilization (Table 2). The fingerprint region displayed the most significant difference between the peaks acquired at predetermined time points. These were observed in between 1700 and 800 cm^−1^. A reduction in the shoulder appearing at 1250 cm^−1^ and 1220 cm^−1^ can be observed; these correspond to the stretching modes of C-O (ether) bonds in the PEEK molecule. The significant spectral features of blank PEEK BL and TL show peaks at 1488 and 1594 cm^−1^ ascribed to phenyl ring stretching bands and 1648 cm^−1^ ascribed to the carbonyl peak. The bending motion of the group (C-C (=O)-C is observed at 1305 cm^−1^. Finally, between 1200 and 600 cm^−1^, phenyl ring C-H deformations are mainly observed in the spectra of the PEEK.

Spectral data collected from Ti scan bodies are represented in Figure 3. Prominent peaks are observed at 2917 and 2851 cm^−1^, which can be assigned to symmetric and asymmetric vibrations of CH and CH_2_ bond stretching. A variation in the band intensities can be observed in both BL and TL spectral data. Another doublet peak formation can be observed at 2040 and 1984 cm^−1^. The peak at 1984 cm^−1^ usually corresponds to carbon–carbon triple bond stretching vibrations. The 2040 cm^−1^ peak is usually assigned to aromatic carbon–carbon double bond stretching vibration. Other Ti-O-C bond stretching vibrations can also be noticed in the range of 1000 to 1500 cm^−1^.

### 3.2. XPS

**Figure 4:**
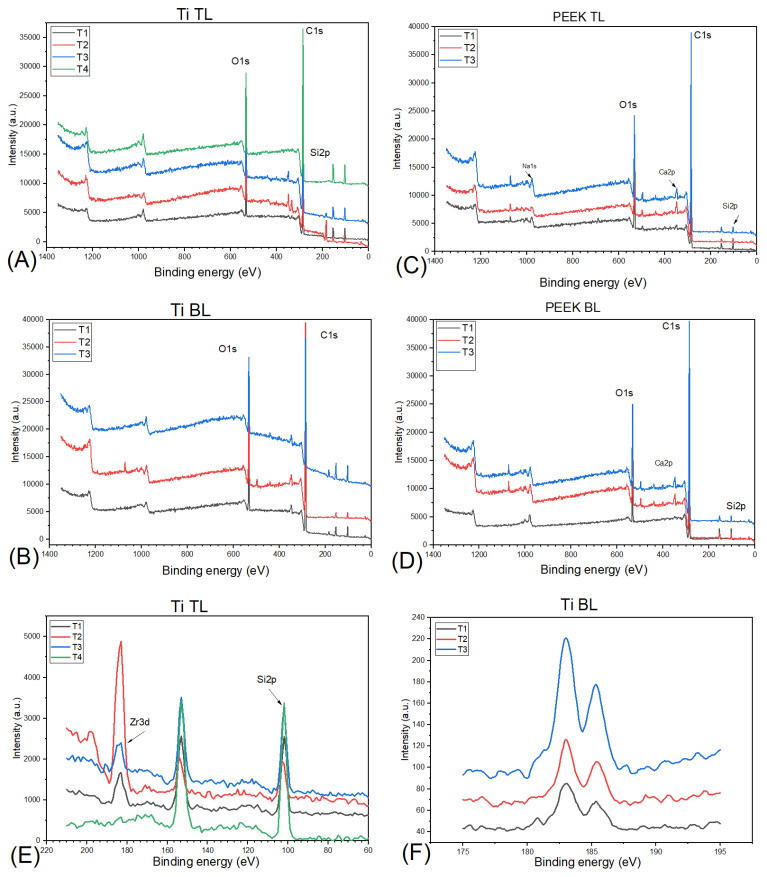
Low-resolution (wide scan) XPS spectral data acquired from of (**A**) Ti tissue level, (**B**) titanium bone level (**C**), PEEK tissue level, and (**D**) PEEK bone level. (**E**,**F**) High-resolution (narrow scan) XPS spectra of the variation in the binding energies of Zr3d and Si2p.

The XPS semi-quantitative analysis of the elemental structure of the surface area of PK, Ti TL, and BL SBs is presented in Figure 4. The binding energy (eV), full width at half maximum (FWHM), and atomic percentages (%) of the elements, detected at each time point, are shown in Table 3 and Table 4, respectively. The low-resolution spectra (wide scan) in Figure 4A–D show strong peaks assigned to carbon (C), oxygen (O), and silicon (Si). The surface chemical composition fluctuated depending on the time points. Interestingly, elemental analysis of the Ti specimens revealed C, O, zirconium (Zr) 3d orbital, and Si 2p electrons. The binding energy of C, O, Si, and Zr remained constant after repeated sterilizations. Variations can be observed in the full width at half maximum. After the third cycle, the atomic percentage of C was slightly higher to the neat and O% decline slightly. These elements varied in their intensities, peak position, and atomic percentages (Table 3). No Ti element was detected in the Ti BL and TL specimens. The oxygen peak was present at 531.78 eV, representing C-O/C=O bonds. The PEEK specimens revealed C, O, and Si. Very low atomic percentages of calcium (Ca2p) and sodium (Na) were identified. The peak area analysis revealed that the carbon and oxygen peaks after sterilization showed broadening. Zr3d consisted of two peaks, corresponding to the electronic configuration of Zr3d_3/2_ (185 eV) and Zr3d_5/2_ (182.4eV).

### 3.3. Surface Roughness

**Figure 5:**
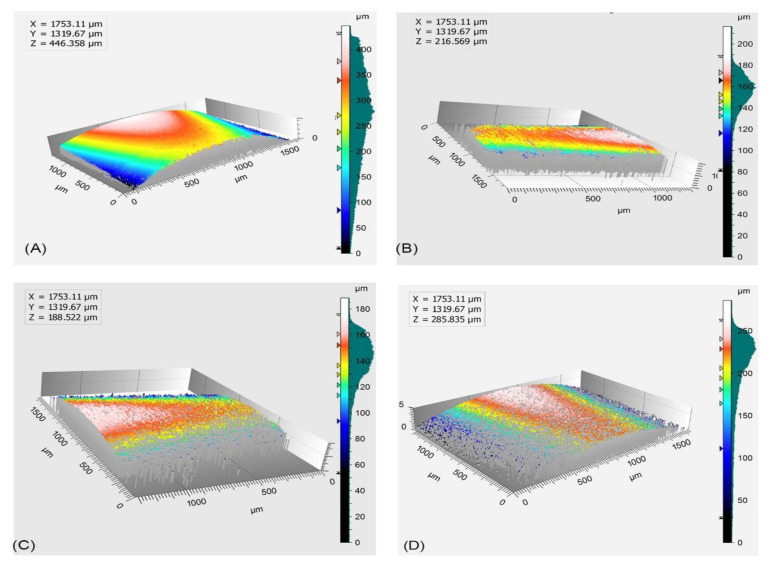
Representative images acquired from the optical profilometer of scan bodies (**A**–**D**) PK BL at baseline and after two sterilizations.

**Figure 6:**
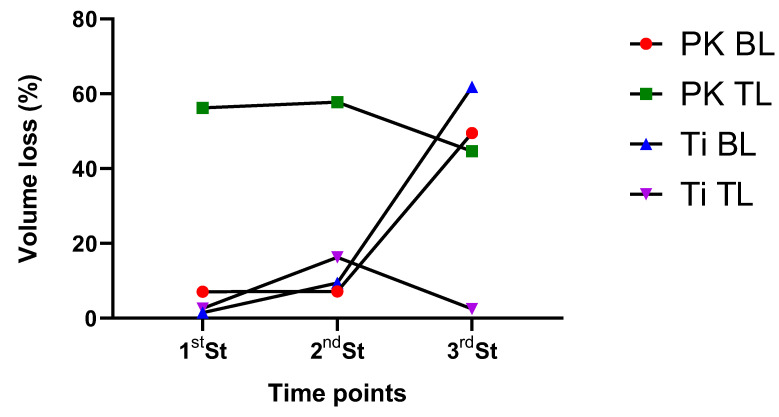
Volume loss percentage (%) of the Sb after 1st, 2nd, and 3rd sterilization cycles.

Optical profilometry was used to investigate the effects of consecutive sterilization on the surface of PEEK and Ti scan bodies. The native surface roughness of samples and the effects of the heat treatments applied are reported in Figure 5 along with representative images of the scan bodies and their profile curve. From the results, the difference between the surface roughness can be noticed. A decrease in the Ra values was observed. A significant difference was observed in between the first and third sterilization for PEEK BL SBs (*p* < 0.05) (Table 5). The results from the volume loss (%) of the SBs are reported in Figure 6. PK BL, Ti BL, and Ti TL showed a <10% volume loss after the first and second sterilization cycles. A higher loss percentage was noted for PK TL (56%) after the first and second cycles. At the end of the third cycle, Ti BL and PK BL showed a 50 to 60% volume loss.

## 4. Discussion

The results of this study indicated that the surface roughness was significantly different after all the three sterilization cycles compared with the baseline values for the tissue-level PEEK scan body; however, the effect was not replicated with the bone-level PEEK scan body, except for the significant differences noted after the third sterilization cycle. Hence, the null hypothesis was rejected for the PEEK TL. The results were mixed for the PEEK BL; thus, the null hypothesis was partly affirmed and partly rejected. As regards Ti SBs, the null hypothesis failed to get rejected for the TL; however, for the BL, there were significant differences in Ra values between baseline and the third sterilization cycle. So, the null hypothesis was neither rejected nor affirmed.

The role of implant scan bodies in digital workflow and the accuracy of digital scans is still not well understood with respect to variables and the overall accuracy. Although they are highly variable in size and shape, the inherent biomaterials used for fabricating these SBs have been either polyether ether ketone (PEEK), titanium (Ti), or their alloys. The aim of the current study was to evaluate the effect of repeated sterilization on the physical and chemical properties of the surface of implant SBs using spectroscopic techniques (Fourier transform infrared and X-ray photoelectron spectroscopy) and surface roughness. The null hypothesis was that the consequent sterilization of PEEK and Ti-Sb did not significantly affect the surface chemical and micromechanical properties. Based on these results, the null hypothesis is accepted.

Fourier transform infrared spectroscopy (FTIR) is a commonly used technique to gain insight into chemical transformations in materials. The need for appropriate chemical characterization of medical devices to assess the materials composition and biological risks can also be found in the ISO 10993-18:2020 standard [8]. A common change in the FTIR spectral profile during polymer degradation is the formation of an absorption band specific to the carbonyl absorption. These carbonyl groups (C=O) absorb the infrared radiation in the wavenumber region of 1540 and 1870 cm^−1^. Another typical alteration during the ageing of PEEK is the formation of a broad hydroxyl group (-OH) absorption at 2800 to 3700 cm^−1^ [9]. Similar spectral variations were observed for the PEEK BL and TL Sb in the current investigation. A significant reduction in the PEEK at 1250 cm^−1^ indicated that the chain cleavage mechanism was occurring generally at the ether bonds as opposed to the carbonyl bonds. The aromatic peaks at 1594 cm^−1^ are very thermally stable and should show the fewest changes. Subtle variations in the peak intensity of the carbonyl bond intensity could be due to thermal degradation. It can be speculated that this thermal degradation occurs by the well-known hydroperoxide mechanism, which is typical for aliphatic polymers [9]. This has also been reported previously by Pascual et al. [10] and Philips et al. [11], whereby they showed that PEEK undergoes degradation by thermal oxidation and thermo-mechanical methods, leading to increase in the chain length due to branching and cross-linking. The significant changes that have been reported previously in the literature take place at the PEEK aromatic ring when treated at high temperatures [12,13]. Although autoclave sterilization is carried out at 121 °C for 15 to 20 min, repeated cycles had a minimal effect on PEEK. PEEK has been frequently reported to have good thermal stability, due to the presence of aromatic groups in its molecular structure [14]. The absence of the Ti element in the XPS scan was indicative of the fact that the surface was covered by a coating. This was evident by the appearance of other elements in the spectra. The spectral profiles of Ti BL and TL Sb revealed that Ti Sb were thermally stable. This thermal stability has been reported previously as well, whereby the temperature of transition of titanium to an anatase phase was noted at 750 °C for 30 min, whereas the autoclave temperature was just 120 °C for 20 min. Even after repeated cycles, the spectral data showed peaks that were coinciding with the peaks noted in the neat spectra. This inherent stability is due to the presence of interstitial elements. There may be a hardening effect associated with heat treatment, as reported earlier by Majchrowicz et al. [15]. The spectral results of the FTIR reveal that there are minimal chemical changes occurring at the Sb surface. Whether they have a significant impact on Sb while being used for the fabrication of implant prosthesis needs further in-depth clinical investigation.

XPS is a powerful tool used to study the surface properties of materials within 5 to 10 nm of the specimen surface along with elemental information [16]. Although reports from the literature suggest that PEEK degradation is an area of interest for investigators in the past [17,18,19], to the best of the authors knowledge no studies have reported the effect of recurrent autoclaving on PEEK, Ti TL, and BL SBs. The XPS results acquired in the current study were in accordance with the previously published PEEK survey scan [18]. The spectroscopic results revealed that there was negligible degradation in PEEK and Ti BL and TL SBs. The implication of these results is that consecutive autoclave sterilization at 120 °C for 15 to 20 min has the least degradation on Ti SBs as compared to PEEK SBs. Usually, the ratio of oxygen to carbon increases significantly if high thermal cycles are used, which is indicative of oxidation, and specimens have higher oxygen contents [20]. This was also observed in the current investigation as the atomic percentages revealed an increase in the oxygen percentage and decrease in the carbon percentage.

Therefore, if further sterilization cycles are intended to reuse SBs, the authors speculate that these cycles should be limited to three to prevent any major chemical alteration in the PEEK Sb especially. A usual XPS survey scan of titanium reveals the very low intensity of Ti2p ½ and Ti2p 3/2 in the region of 450 to 470 eV. These peaks are assigned to the oxidation states of titanium. Moreover, they have been reported to have low intensities and are in close proximity to each other [21]. The absence of these peaks could be due to the presence of coating on SBs. Titanium implants have been reported to be strongly discolored after autoclaving.

Surface roughness significantly favors bacterial attachment and biofilm formation and facilitates its growth [22]. Therefore, dental restorations are preferred to maintain a smooth surface. Evidence from the literature suggests that two methodologies have been adopted to analyze surface roughness: a two-dimensional method using a contact profilometer with a diamond tracing stylus, or a method using a non-contact three-dimensional optical profilometer. Amongst these two, the use of the non-contact 3D profilometer has been extensively reported [22,23,24]. Like any other synthetic polymers, PEEK when subjected to repeated heating cycles can experience changes in its roughness. These can be attributed to several factors such as the material’s thermal stability, the specific heat-treatment process, and the length of the treatment. Some of the reported consequences of heat treatments are related to softening and flow, crystallinity [25], oxidation, stress relaxation, and thermal expansion. PEEK has a high glass transition temperature (143–150 °C) [26]. Using autoclave at 120° might lead to the softening of the polymer and potentially cause the biomaterial to slightly deform or flow at the surface. PEEK can also crystalize to varying degrees depending on the processing condition. Similarly, repeated or prolonged heating at elevated temperatures can lead to oxidation.

In this study, a single-scan body was used for each design for both materials and tested for various parameters after repeated use and sterilizations. The choice of using a single-scan body was based on previous similar studies that had evaluated the accuracy of the digital impressions with SBs after repeated use. Since there were no additional factors assessed in this investigation, the requirement for testing more scan bodies of each type was deemed unnecessary.

Heating cycles can also affect the titanium surface as well. These may be dependent on multiple factors, including the titanium alloy composition, heating duration, and environmental conditions. Generally, titanium is resistant to oxidation and maintains its physical properties at high temperature. However, due to the alloy composition as reported by XPS data in the current study, a decrease in the roughness was observed. Previous reports suggest that autoclaving implants results in lower Ra values, whereas ultraviolet sterilization increases the surface roughness, which indicates that there may be steam-borne contaminants being deposited during autoclaving [27]. Interestingly, after repeated sterilization cycles the surface roughness of Ti BL and TL SBs decreased significantly, supporting the observations made previously by Park et al. [27] and Smith et al. [28] with autoclaving-altered Titanium alloy substrates. Total-volume-loss analysis generated interesting results. Whilst, PK BL, Ti BL, and Ti TL showed a gradual volume loss which could be attributed to the initial surface loss, PK TL showed a much higher volume loss immediately after the first sterilization cycle. This higher surface loss could be due to the degradation of the thin surface layer after the first exposure to autoclaving, as thermal ageing of polymers is commonly manifested by the formation of an oxidation layer; embrittlement; or discoloration [29]. The PEEK surface may also experience thermal stresses that cause micro-cracks or sites for future degradation. Subsequent cycles may cause incremental loss of layers. Therefore, it is speculated that the degradation behavior of PK Sb is a surface-driven process.

Future investigations need to analyze the scan bodies after a higher number of sterilization cycles to identify the changes in surface characteristics better. Whilst FTIR and XPS were useful techniques to detect certain chemical changes on the surface, the absence of spectra at some time points should be viewed as a limitation inherent to the technique, particularly when dealing with subtle surface modifications. Also, the effect of multiple sterilization cycles on the bulk properties of SBs needs to be assessed further using advanced characterization techniques, like scanning electron microscopy and transmission electron microscopy. Prospective clinical studies using intra-oral scanners are required to confirm the accuracy of the prosthesis fit obtained using reused scan bodies after repeated sterilization cycles.

## 5. Conclusions

XPS and FTIR analyses reveal that autoclave sterilization minimally affects the chemical properties of both PEEK and titanium SBs. However, PEEK’s surface roughness showed changes with repeated heating cycles. The careful management of sterilization cycles is recommended for the prolonged reuse of PEEK SBs.

## Figures and Tables

**Table 1:** Types of Dental implant scan bodies and manufacturer details.

Material	PEEK	PEEK	Titanium	Titanium
**Manufacturer**	**Straumann^®^** **Basel Switzerland**	**Straumann^®^** **Basel Switzerland**	**Straumann®** **Basel Switzerland**	**Straumann®** **Basel Switzerland**
**Type**	Tissue Level	Bone Level	Tissue Level	Bone Level

**Table 2:** Main spectral changes observed in PEEK BL and TL after sterilization from 600–1800 cm^−1^.

Wavenumber (cm^−1^)	Spectral Changes and Identification
1741	Fluorenone peak formation
1277 and 1305	Two bands pertaining to high frequency of the ether peak at 1219 cm^−1^
1100	Alterations in the diphenyl ether bonds
1185	Disappearance of peak of diphenyl ether group
837	Changes in the aromatic rings
926	Increased intensity after sterilization

**Table 3:** XPS semi-quantitative analysis of the data acquired from titanium bone level (Ti BL) and titanium tissue level (Ti TL). BE = binding energy (eV); FWHM = full width at half maximum (eV); and % = atomic percentage in the order of BE, (FWHM), %.

	Neat (Pristine)		1st ST	
Elements	Ti BL	Ti TL	Ti BL	Ti TL
C1s	284.66, (1.3), 76.66	284.67, (1.11), 79.67	284.63, (1.09), 77.02	284.67, (1.11), 79.67
O1s	531.96, (1.76), 15.79	530.88, (1.61), 14.66	531.78, (1.89), 17.21	531.88, (1.61), 14.66
Si2p	101.87, (1.64), 7.24	101.9, (1.49), 4.84	101.9, (1.52), 3.9	101.9, (1.49), 4.84
Zr3d	183.5, (1.68), 0.31	182.58, (1.24), 0.83	182.26, (1.24), 1.87	182.58, (1.24), 0.83
Ca2p	-	-	-	-
	**2nd ST**		**3rd ST**	
	**Ti BL**	**Ti TL**	**Ti BL**	**Ti TL**
C1s	284.62, (1.49), 71.03	284.6, (1.32), 79.28	284.62, (1.49), 81.95	284.60, (1.32), 79.28
O1s	531.92, (1.87), 17.9	531.8, (1.71), 13.95	531.92, (1.87), 11.50	531.80, (1.71), 13.95
Si2p	101.87, (1.79), 9.2	101.76, (1.57), 5.2	101.87, (1.79), 5.33	101.76, (1.57), 5.20
Zr3d	182.84, (1.47), 0.53	182.84, (1.25), 0.22	182.84, (1.47), 0.53	182.84, (1.25), 0.22
Ca2p	347.02, (2.06), 1.34	347.02, (1.6), 1.36	347.29, (2.06), 1.22	347.02, (1.60), 1.36

**Table 4:** XPS semi-quantitative analysis of the data acquired from on PEEK bone level (PK BL) and PEEK tissue level (PK TL). BE = binding energy (eV); FWHM = full width at half maximum (eV); and % = atomic percentage in the order of BE, (FWHM), %.

	Neat (Pristine)		1st ST	
**Elements**	**PK BL**	**PK TL**	**PK BL**	**PK TL**
C1s	284.61, (1.11), 81.05	284.65, (1.1), 83.08	284.61, (1.11), 81.05	284.69, (1.04), 78.3
O1s	531.6, (1.79), 13.15	531.69, (1.7), 12.56	531.6, (1.79), 13.15	1531.88, (1.71), 5.11
Si2p	101.79, (1.55), 2.29	101.83, (1.36), 2.10	101.79, (1.55), 2.29	101.94, (1.39), 5.03
Na1s	1071.29, (1.71), 0.75	1071.42, (1.73), 0.21	1071.29, (1.71), 0.75	1071.13, (1.26), 0.68
Ca2p	347.04, (1.46), 1.59	347.12, (1.44), 1.78	347.04, (1.46), 1.59	347.02, (1.41), 0.87
Zn2p	1021.7, (1.61), 0.39	-	284.61, (1.11), 81.05	-
	**2nd ST**		**3rd ST**	
	**PK BL**	**PK TL**	**PK BL**	**PK TL**
C1s	284.68, (1.28), 79.81	284.67, (1.14), 84.4	284.68, (1.28), 79.81	284.67, (1.1.4), 84.40
O1s	531.98, (2.06), 15.83	531.61, (1.80), 11.56	531.98, (2.06), 15.83	531.61, (1.80), 11.56
Si2p	102.03, (1.73), 2.36	101.86, (1.80), 1.9	102.03, (1.63), 2.36	101.86, (1.43), 1.90
Na1s	1071.10, (1.73), 0.78	1071.22, (1.94), 0.73	1071.10, (1.73), 0.78	1071.22, (1.94), 0.73
Ca2p	346.98, (1.68), 1.21	347.06, (1.53), 1.41	346.98, (1.68), 1.21	347.06, (1.53), 1.41
Zn2p	-	-	-	-

**Table 5:** Ra values taken at different time points. T1 (baseline), after first sterilization (T2), second sterilization (T3), and third sterilization (T4). Values shown are mean ± SD. Significant differences observed.

	T1	T2	T3	T4
PK BL	3.40 ± 1.00	3.16 ± 1.38 (0.70)	3.66 ± 0.68 (0.43)	1.71 ± 0.33 (0.006)
PK TL	3.52 ± 0.72	1.54 ± 0.56 (<0.001)	1.48 ± 0.18 (<0.001)	1.95 ± 0.68 (<0.001)
Ti BL	4.39 ± 0.80	4.46 ± 0.61 (0.83)	3.97 ± 0.70 (0.36)	1.67 ± 0.49 (<0.001)
Ti TL	2.64 ± 0.70	2.76 ± 0.51 (0.68)	2.21 ± 0.62 (0.25)	2.57 ± 0.32 (0.82)

## Data Availability

The raw data supporting the conclusions of this article will be made available by the authors on request.
